# Code Telestroke: On the Necessity of Formalizing Training in Telestroke for Future Vascular Neurologists

**DOI:** 10.1212/NE9.0000000000200246

**Published:** 2025-08-26

**Authors:** Amanda L. Jagolino-Cole, Christina Mijalski, Amy Katherine Guzik

**Affiliations:** 1 Department of Neurology, McGovern Medical School at the University of Texas Health Science Center at Houston;; 2 Department of Neurology and Neurological Sciences, Stanford Medicine; and; 3 Department of Neurology, Wake Forest University School of Medicine, Winston-Salem, NC.

Neurologists have used telestroke to reach neurologically underserved patient populations for decades.^1^ Stroke care through telemedicine leads to accurate thrombolytic decision making, safe patient outcomes, appropriate triage and transfer, and cost-effectiveness, improving resource utilization within a health care system.^2^ Acute stroke guidelines recommend telestroke consultation to provide timely guidance for high-stakes decision making and support community hospitals for stroke center certification coverage requirements.^3^ Telestroke education for stroke fellows improves efficiency and decision-making skills, is safe, and can align with Accreditation Council for Graduate Medical Education (ACGME) core competencies.^4^ Yet, current expectations for telestroke training lack a standardized structure, with limited guidance on monitoring of metrics or methods of training especially for under-resourced programs. Furthermore, there is no current standard for the minimum requirements that neurologists must meet for independent practice of telestroke. With the prevalence of remote patient care in clinical stroke practice, formalized telestroke education should be a required component of vascular neurology training programs, endorsed and directed by governing bodies including the ACGME and the American Board of Psychiatry and Neurology (ABPN).

Telestroke training requires infrastructure and resources, and cooperation from network telestroke hospitals. Multiple groups including national professional organizations (e.g., ACGME and ABPN) and local institutions (e.g., neurology departments, credentialing, billing, and compliance) must collaborate to successfully enact telestroke training plans and ensure that program learning agreements, faculty supervision requirements, and patient safety metrics are met. Fiscal responsibility among stakeholders within a network and consistent support for reimbursement add to the complexity in assuring that faculty educators have sufficient time and space to teach fellows. For vascular neurology fellowship programs without an established telestroke network, complexity is higher, although collaboration between programs and “virtual electives” could bridge this divide.^4^ Without national telestroke training standards, programs cannot advocate for resources needed to provide telestroke education to trainees.

Clinicians have provided telestroke care for years without formal training requirements in remote patient care. However, the complexities of providing efficient and effective stroke care remotely have expanded with updated standards of stroke care, increasing time windows and therapeutic interventions, and an evolving understanding of stroke systems of care. Although 1 Vascular Neurology Milestone was incorporated in ACGME fellowship requirements in 2021, this does not encompass the intricacy of managing patients through telestroke, and further intentional, focused training is indicated along with educational benchmarks.^4^

Telestroke became the standard of care^2,3^ to reach patients with limited access to expert and timely acute stroke care, despite lack of clarity on reimbursement for telestroke and a dearth of supervision and educational requirements. We now apply the standardized program requirements to remote care, under the assumption that telestroke can be performed without formal training. Yet, we also know that telestroke-specific training and repetition in this care environment lead to more efficient treatment decisions and familiarity with telestroke, not only for vascular neurology fellows but even earlier at the neurology resident level.^4,5^ While telestroke has become standard clinical practice, we have sped past the formalization of training and milestones, leaving at least some vascular neurology trainees unprepared or underprepared for efficient remote patient care when starting independent practice, often necessitating additional training by hiring telestroke programs. Telestroke competencies reverberate ACGME and ABPN educational goals including increasing medical knowledge, refining patient care skills, and applying expertise to expanding clinical settings.^4^ For the benefit of our trainees and the remote patients they care for, structured, formalized training in telestroke should be a required component of vascular neurology training programs to fully train competent clinicians with the comprehensive skills applicable to subspecialty fellowship graduate medical education.
